# Extracellular release in the quality control of the mammalian mitochondria

**DOI:** 10.1186/s12929-023-00979-3

**Published:** 2023-10-08

**Authors:** Kuei-Hsiang Pan, Hung Chang, Wei Yuan Yang

**Affiliations:** 1 Institute of Biological Chemistry, Academia Sinica, Taipei, Taiwan; 2 Institute of Biochemical Sciences, College of Life Sciences, National Taiwan University, Taipei, Taiwan

**Keywords:** Extracellular vesicles, Mitochondria, Organelle quality control, Autophagy, Mitophagy

## Abstract

Mammalian cells release a wealth of materials to their surroundings. Emerging data suggest these materials can even be mitochondria with perturbed morphology and aberrant function. These dysfunctional mitochondria are removed by migrating cells through membrane shedding. Neuronal cells, cardiomyocytes, and adipocytes send dysfunctional mitochondria into the extracellular space for nearby cells to degrade. Various studies also indicate that there is an interplay between intracellular mitochondrial degradation pathways and mitochondrial release in handling dysfunctional mitochondria. These observations, in aggregate, suggest that extracellular release plays a role in quality-controlling mammalian mitochondria. Future studies will help delineate the various types of molecular machinery mammalian cells use to release dysfunctional mitochondria. Through the studies, we will better understand how mammalian cells choose between intracellular degradation and extracellular release for the quality control of mitochondria.

## Background

Mitochondria carry out beta-oxidation and oxidative phosphorylation, two programs that elicit reactive oxygen species (ROS) formation. The ROS produced can oxidize molecules within mitochondria and cause mitochondrial damage. Mammalian cells, therefore, need to actively quality control their mitochondria. Numerous intracellular mitochondrial quality control mechanisms have been discovered for mammalian cells in the past two decades. One well-studied mechanism is mitophagy, the process by which damaged or dysfunctional mitochondria are selectively targeted for degradation by the autophagy pathway [1, 2]. On the other hand, cells can also segregate defective portions of the mitochondria for removal through the generation of mitochondrial vesicles [3, 4]. Furthermore, mammalian cells possess molecular chaperones and proteases to control mitochondrial protein quality. These chaperones and proteases help ensure that individual mitochondrial proteins are either correctly folded or cleared away [5]. Finally, besides removing damaged mitochondria, cells also activate mitochondrial biogenesis to synthesize new mitochondria to replace the damaged ones [6].

While most studies on mitochondrial quality control focus on intracellular pathways, one has to wonder whether the extracellular release of mitochondrial components also plays a significant role in clearing away damaged mitochondria. After all, mammalian cells are known to be capable of releasing materials to their surroundings. For example, cells can release individual molecules, including hormones, neurotransmitters, and enzymes, to modulate their environments. Moreover, it is widely observed that mammalian cells dump large and complex cargos, up to the scale of entire organelles, into their surroundings.

## Ways of releasing materials into the extracellular space

Cells can release materials in a non-membrane-bound ("free") form. For example, materials can be sorted into secretory vesicles for their release into the extracellular environment [7]. This is primarily how bio-active molecules such as hormones and neurotransmitters are shipped to the extracellular space. On the other hand, mammalian cells can also release cargos in the membrane-enclosed form (extracellular vesicles; EVs). EVs are defined as "lipid bilayer-enclosed extracellular structures" [8] and exist in a wide range of sizes (from ~ 30 nm up to µm-sized). These membrane-bound structures can be generated through the outward budding of the plasma membrane, resulting in the formation of what are termed the ectosomes. The structures can also form through the fusion of multivesicular bodies (MVBs; endosome-derived vesicles) to the plasma membrane, resulting in the release of the intraluminal vesicles (ILVs) from MVBs, which are termed the exosomes.

The existence of EVs in mammalian tissues or fluids was long described several decades ago, but in many cases, the precise functions of EVs remain obscure. EVs were initially considered to represent only cell debris and lack specific biological functions. It is now recognized that EVs can carry a complex mixture of bioactive contents. Proteins, DNA, RNA, and lipids can all reside within EVs. EVs can be engulfed by surrounding cells through processes such as clathrin-mediated endocytosis, phagocytosis, and micropinocytosis. Cargos within EVs can also be injected into target cells through direct EV-plasma membrane fusion [9]. The proposed functions of EVs now include intercellular communication, and the elimination of unnecessary or excess intracellular components, as cells can use EVs to deliver intracellular materials into the extracellular space [10]. Recent studies have indicated that these functions of EVs might take part in several physiological contexts and pathogenesis of diseases, including immune response, skin pigmentation, cancer progression, and neurodegeneration disease [11–16]. Evidence now suggests that the released vesicles under these physiological circumstances can even contain entire organelles.

## Examples of organelle release

Components of organelles can be released bare and in membrane-bound structures. An example of the former is the observation that lysosomes can fuse directly with the plasma membrane (PM). This fusion leads to the release of lysosomal contents to the cellular surrounding. To achieve this, lysosomes move from the perinuclear region toward the PM, where Ca^2+^ transients trigger lysosome-PM fusion. This process is termed lysosomal exocytosis [17, 18]. Lysosomal exocytosis acts to facilitate membrane repair following PM-wounding. Acid sphingomyelinase released during lysosomal exocytosis help trigger the internalization of wounded PM to allow cellular repair following injury [19, 20]. In addition, immune cells release lysosomal hydrolases through lysosomal exocytosis to restrict the propagation of intruding pathogens [21, 22].

**Fig. 1:**
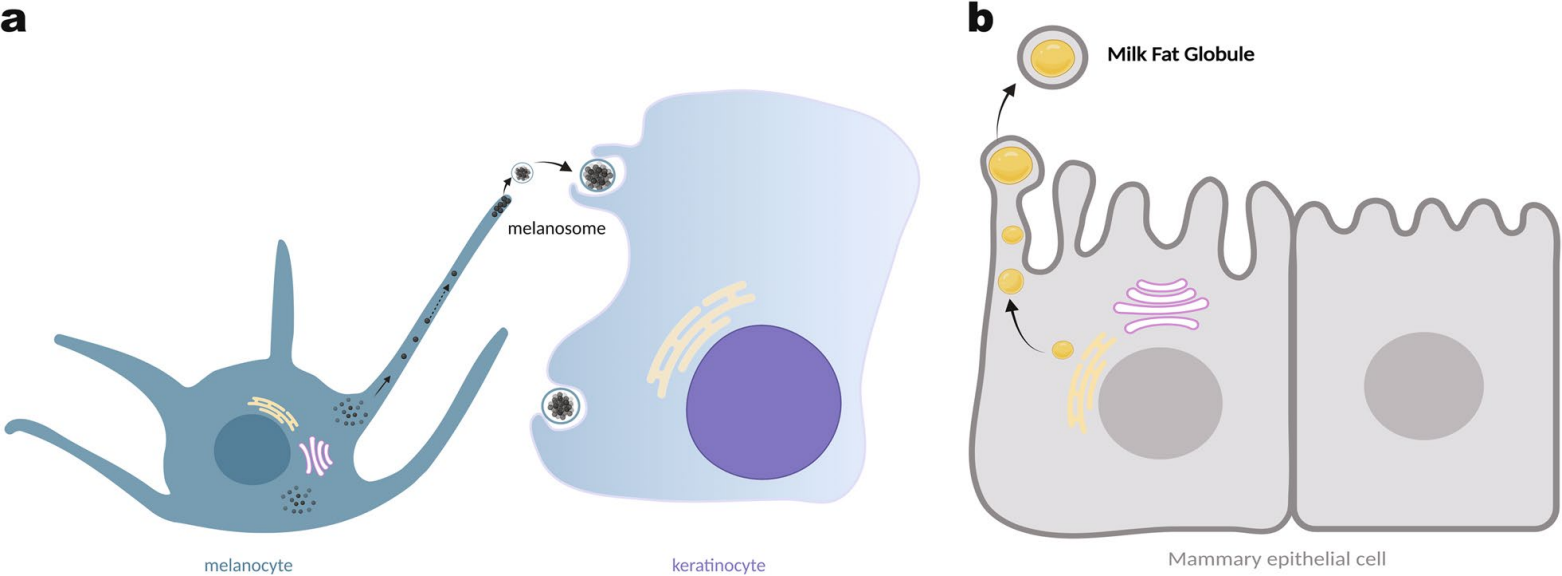
Organelle Extracellular Release. **a**. Melanosome extracellular release: melanosomes, derived from multivesicular bodies (MVBs), are transported to the tip of melanocytes’ dendrites and released. The released melanosomes are subsequently taken up by neighboring keratinocytes. Yellow: Endoplasmic reticulum (ER); Red: Golgi apparatus **b**. Lipid droplet extracellular release in mammary epithelial cells: lipid droplets, originating from the endoplasmic reticulum (ER), bud off and fuse with the plasma membrane, forming milk fat globules (MFGs). Figure created with BioRender.com

Examples of organelle parts released as membrane-bound cargos are also frequently observed. Two types of EV-mediated organelle release have been extensively studied, with their functions well-established. First, in the human skin, melanocytes produce melanin and transfer it to adjacent keratinocytes to regulate skin color and protect DNA in these epidermal cells from solar UV damage. Melanin is packaged in a specialized organelle in melanocytes called the melanosome. It has been shown that the extracellular release of melanosomes is responsible for melanin delivery into keratinocytes (Fig. 1a). Previous literature has revealed that the melanosomes are transported toward and assembled at melanocytes dendrite, followed by the budding and shedding of a plasma membrane-enclosed package containing multiple melanosomes [11, 12, 15]. The adjacent keratinocytes then phagocytose this melanosome package to complete the melanin transfer process.

The second involves the release of lipid droplets from lactating mammary glands (Fig. 1b). The lipid droplet fraction of milk is crucial for newborns since it supplies lipid sources for their development. During milk-lipid secretion, the plasma membrane of mammary epithelial cells envelops intracellular lipid droplets and then buds off from the apical plasma membrane, forming milk fat globules (MFG) [8, 23, 24]. The extracellular release of melanosomes and lipid droplets described above both rely on the formation of ectosomes.

Meanwhile, reports also indicate the use of exosomes in driving organelle release. Previous literature suggests that ER can be engulfed into MVBs and reside within the ILVs of MVBs. VAMP7-mediated fusion of MVBs with the plasma membrane result in the release of ER fragments within exosomes. While the exact function of ER-carrying exosomes remains obscure, this release process is thought to help expand the plasma membrane and facilitate neurite outgrowth [25, 26]. This process is termed secretory reticulophagy (SERP).

## Extracellular release of mitochondria

Like other organelles, mitochondria can also be released by mammalian cells. The mitochondria possess a number of characteristics that facilitates their detection within EVs or the extracellular space. Mitochondria exhibit a distinct membrane topology, making them recognizable under electron microscopy even when fragmented. In addition, mitochondria possess their own DNA, allowing for another handle that can be used for detection in fluorescence imaging or biochemical-based analyses. These properties have allowed the discovery of cellular settings that display mitochondrial extracellular release. The released content can be functional mitochondria or bio-active mitochondrial components for modulating nearby cells. Emerging data also suggest that damaged mitochondria can be selectively picked out and shipped to the extracellular space, presumably for quality control.

### Mito-release for immune-activation

Cells release mitochondria or mitochondria-containing EVs to evoke an immune response in target cells [27–31]. For example, monocytic cells release free mitochondria and microvesicles with mitochondrial contents upon lipopolysaccharide (LPS) stimulation [30]. These released mitochondria and microvesicles induce type I IFN (interferon) and TNF (tumor necrosis factor) dependent responses, including the induction of ICAM-1 (intercellular adhesion molecule), VCAM (vascular cell adhesion molecule) mRNA expression level as well as IL-8 production in endothelial cells [30]. Furthermore, activated platelets could release mitochondria, again either as free organelles or within membrane-encapsulated microparticles, into the blood plasma [27]. Blood plasma sPLA2-IIA (Secreted phospholipase A2 group IIA), an endogenous phospholipase specific for bacteria, then hydrolyzes lipids on the mitochondrial membrane to release various lysophospholipids, free fatty acids, and mtDNA to promote leukocyte activation [27]. It has also been shown that activated microglia release fragmented, dysfunctional mitochondria to activate astrocytes, leading to the propagation of neuroinflammation [28].

That mitochondrial release can lead to immune modulation is thought to arise from proinflammatory signals that reside within mitochondria. Unlike nuclear DNA, the mitochondrial genome is circular, free of histones, and shows limited methylation on CpG islands. These characteristics of mitochondrial DNA share resemblances with the bacterial genome, which act as potential agonists of immune response once present in the cytosol or the extracellular environment [32]. Aside from mtDNA, mitochondrial components such as N-formyl peptides, cardiolipin, and cytochrome C also display proinflammatory properties.

### Mito-release for boosting mitochondrial functions in recipient cells

Can the extracellular release of mitochondria, or components thereof, improve mitochondrial functions in recipient cells? Can the released material even be functional, and capable of integrating into the existing mitochondrial network of the recipient cells upon internalization?

Both neuronal cells and mesenchymal stem cells (MSCs) have been found to release EVs containing functional mitochondria [33–37]. Neuronal cells release mitochondria with intact ultrastructure and membrane potential, which have been shown to alleviate stress in recipient cells [33, 34]. Similarly, MSCs have also been observed to release functional mitochondria into the extracellular space, as demonstrated by co-culture studies and mitochondrial membrane potential-dependent dye staining [35–37]. Upon uptake by recipient cells, these MSC-released mitochondria can fuse with existing mitochondrial networks, indicating their functional capacity [37]. This transfer of functional mitochondria has been linked to enhanced bioenergetics, as evidenced by increased oxidative phosphorylation and ATP production in recipient cells [35–37]. These properties may offer a potential explanation for the observed health benefits associated with MSC-based therapies.

### Mito-release for quality control

#### Mitochondrial release during cell migration

Cells undergoing migration can generate membrane protrusions and release EVs known as migrasomes, which contain cellular components that accumulate and pinch off from the tip of the protrusions [38]. Studies indicate that migrasomes are oftentimes assembled within membrane microdomains enriched with tetraspanins and cholesterol. The presence of tetraspanins and cholesterol on these microdomains is believed to enhance membrane rigidity, facilitating the formation of a bulging membrane structure necessary for migrasome development [39].

**Fig. 2:**
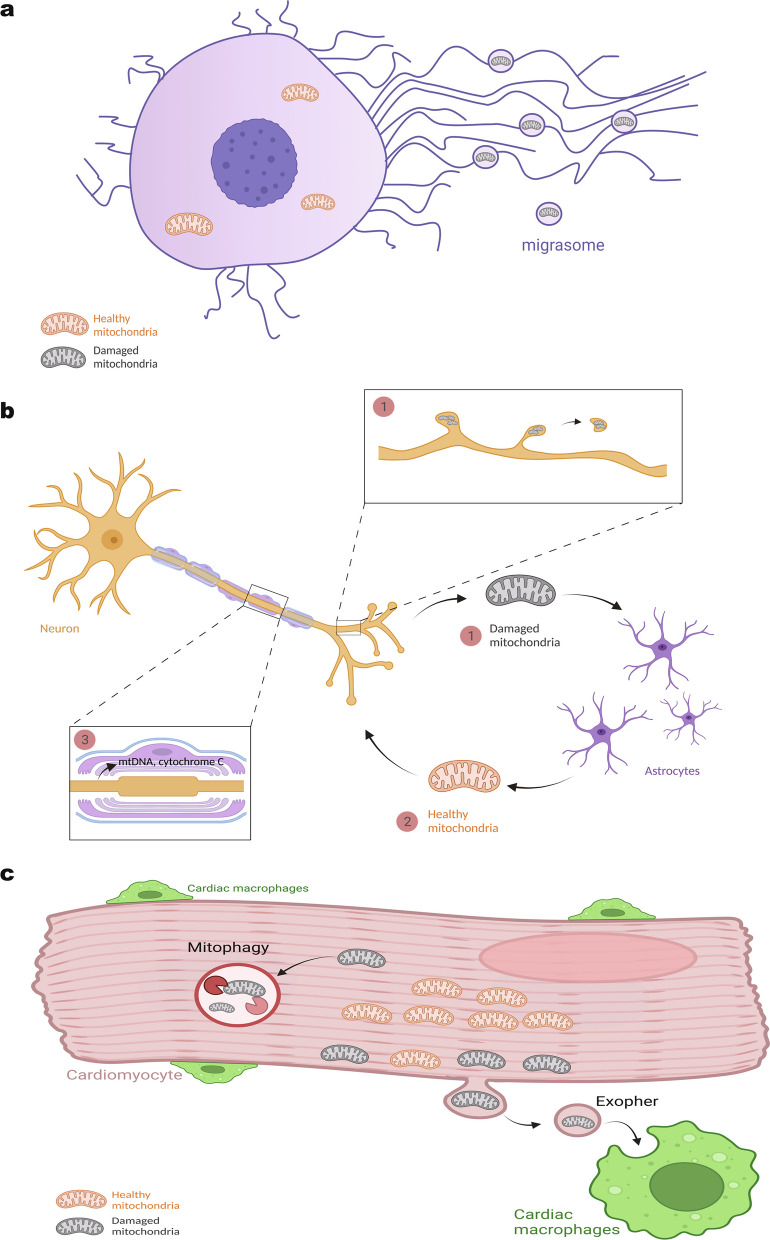
Mitochondria Extracellular Release as a Quality Control Mechanism. **a**. Damaged mitochondria are selectively transported to migrasomes through retraction fibers and released. **b**. (1) Neurons are able to deliver damaged mitochondria to adjacent astrocytes for their degradation. (2) Meanwhile, astrocytes can deliver functional mitochondria back to neurons in order to compensate for malfunctioning mitochondria. (3) When neurons suffer from undesirable conditions, Schwann cells can release mtDNA and cytochrome C as warnings to inform neurons of potential danger and degeneration. **c**. Mitochondria quality control in cardiomyocytes occurs through two pathways. One, cardiomyocytes can release damaged mitochondria to cardiac macrophage through exophers for degradation. Two, the damaged mitochondria can also be degraded by mitophagy. Figure created with BioRender.com

Recent studies have shown that when migrating cells are treated with low concentrations of the oxidative phosphorylation uncoupler carbonyl cyanide 3-chlorophenylhydrazone (CCCP at 2 µM), dysfunctional mitochondria can be released into the extracellular space through migrasomes (Fig. 2a). The mitochondria residing in these migrasomes display a swollen morphology, generate higher than normal levels of reactive oxygen species, and possess lower mitochondrial membrane potential when compared to mitochondria in the cytoplasm [40]. Mechanistically, it has been found that migrasome-released mitochondria recruit less of the motor protein dynein but more KIF5B, leading to their selective accumulation at the cell periphery for extracellular release. This selective release of dysfunctional mitochondria suggests that mitocytosis (a term used to describe the migrasome-mediated mitochondria release) is a quality control mechanism for mitochondria. Studies have demonstrated that mitocytosis is required to maintain mitochondrial respiration and membrane potential in neutrophils and is necessary for macrophage differentiation in vivo. Interestingly, higher concentrations of CCCP (10 µM) lead to intracellular degradation of mitochondria through mitophagy instead of mitocytosis. Future studies will better define the interplay between mitocytosis and mitophagy in the quality control of mitochondria in migrating cells.

#### Horizontal mitochondrial transfer in the neuronal system

Numerous studies indicate that neuronal mitochondria can be transferred into glial cells [41–45]. Some of these studies suggest that EVs facilitate this mitochondrial delivery. For instance, 3D images of mouse retinal ganglion cell axons obtained through serial block-face scanning electron microscopy reveal that axonal mitochondria can be sent into the extracellular space through the formation of protrusions [45]. Furthermore, when retinal ganglion cell mitochondria were labeled with the tandem-fluorescent mitochondrial marker MitoEGFPmCherry, it was discovered that some of these mitochondria are eventually targeted to the lysosomes of nearby astrocytes. Subsequent examination using the TUNEL assay revealed that the mitochondrial DNA from these expelled mitochondria undergoes degradation within astrocytes. Collectively, these findings suggest that neuronal mitochondria can be released by axons and subsequently targeted to neighboring astrocytes for degradation. The presence of disease-promoting mutations in Optineurin, a known mitophagy-related protein, enhances this mitochondrial degradation activity [46]. This transcellular mitochondrial degradation activity has been termed "transmitophagy" (Fig. 2b).

The release of neuronal mitochondria can be amplified when challenged with various mitochondrial toxins. It is well-established that mitochondrial toxins can induce the release of mitochondrial components from neurons and affect nearby glial cells [47]. For instance, administration of 6-hydroxydopamine (6-OHDA) to mice leads to degeneration of nearby dopaminergic neurons, resulting in the shedding of micron-sized spherical particles (spheroids) from their axons [44]. These spheroids accumulate damaged mitochondria positive for PINK1/Parkin markers, which eventually enter the lysosomes of neighboring astrocytes. Similarly, employing optogenetic techniques to induce ROS-stress in mitochondria [48] within cone photoreceptor cells leads to the delivery of these ROS-stressed mitochondria into the lysosomes of Muller glia cells [42]. Electron microscopy-based analysis revealed that some of the mitochondria may have been transported from cone photoreceptors to Muller glia cells through budding of the plasma membrane. Both observations suggest that damaged mitochondria are released extracellularly and contribute to the occurrence of neuron-to-glia transmitophagy.

Conversely, it has also been demonstrated that astrocytes are capable of packaging mitochondria into EVs [34]. Electron microscopy studies have shown that cultured astrocytes release EVs containing mitochondria with intact cristae structures. These astrocyte-released mitochondria can be stained with mitochondria dyes that are dependent on membrane potential, indicating their functionality. When astrocytes are co-cultured with neuronal cells, the EVs released by astrocytes, containing functional mitochondria, can be taken up by neuronal cells and even fused into the neuronal mitochondrial network. Consequently, these EVs have been observed to enhance neuronal viability. For example, when astrocyte-released EVs containing functional mitochondria were administered into the brains of mice subjected to focal cerebral ischemia, they upregulated cell survival signals in mouse brain neurons. The observation that EVs can mediate neuron-to-glia transmitophagy of damaged mitochondria, as well as the delivery of functional mitochondria from glial cells back to neurons to improve neuron survival, suggests that EV-mediated mitochondrial exchange in the neuronal system may serve as a mitochondrial quality control mechanism.

#### Horizontal mitochondrial transfer in the heart

Research findings indicate that EV-mediated transmitophagy may play a role in facilitating mitochondrial quality control in the heart, similar to what was observed in the neuronal system (Fig. 2c). Studies have shown that the removal of cardiac-resident macrophages (cMacs) in mice leads to perturbations in mitochondrial qualities within cardiomyocytes. In the absence of cMacs, mitochondria in cardiomyocytes exhibit reduced cristae density and a decreased capacity for ATP production. Conversely, it has been observed that cardiomyocytes release micron-sized EVs, termed exophers, which contain structurally perturbed mitochondria. This process of EV-mediated mitochondrial disposal is upregulated when the mitochondria within mouse cardiomyocytes are subjected to stress, such as the administration of isoproterenol. Through the use of a pH-sensitive, mitochondria-targeted fluorescent reporter called mtKeima, researchers have determined that cardiomyocyte-derived extracellular mitochondria are engulfed by cMacs for lysosomal degradation. Taken together, these findings suggest that the cardiomyocyte-to-cMacs transmitophagy contributes to the quality control of mitochondria in the heart [49].

The ability of cMacs to remove damaged mitochondria from the heart implies a potential therapeutic value of these cells in mitigating heart-related disorders. Supporting this expectation, studies have demonstrated that the transplantation of cMacs into the pericardial space can prevent sepsis-induced cardiomyopathy in mouse models [50]. This beneficial effect is thought to be mediated by cMacs' ability to remove dysfunctional mitochondria from cardiomyocytes, thereby preserving cardiac function.

#### Horizontal mitochondrial transfer by the adipose tissue

Ample observations indicate that adipocytes undergo EV-mediated release of oxidized mitochondrial components. When brown adipose tissue (BAT) is exposed to cold stress, a condition known to stimulate BAT mitochondrial ROS production to sustain thermogenesis [51, 52], there is a notable increase in the release of specific mitochondrial proteins. These proteins, such as the pyruvate dehydrogenase E1 subunit beta (PDHβ) and pyruvate decarboxylase, are transported via larger EVs (approximately 350 nm in diameter) [53]. Remarkably, the proteins released through these EVs exhibit signs of oxidation, including the presence of 4-Hydroxynonenal (4-HNE) and the formation of carbonyl protein adducts. Analysis of the released PDHβ reveals a higher degree of cysteine oxidation compared to its intracellular counterpart. Adipose tissue-resident macrophages play a crucial role in clearing these EVs induced by cold stress, thus preventing dysfunction in BAT.

In another scenario, mimicking obesity-associated impairment of mitochondria in the adipose tissue through mitochondrial ferritin (FtMT) overexpression in mice also prompts adipocytes to release smaller EVs such as exosomes [54]. The small EVs (sEV) are enriched with selective mitochondrial proteins including voltage-dependent anion channel (VDAC), heat shock protein 60 (HSP60), and cytochrome c oxidase subunit 4l1 (COXIV). Functional assessments of isolated sEVs from FtMT mice reveal that they are sub-competent in oxygen consumption when supplied with ADP, accompanied by elevated levels of carbonyl protein adduct formation. This leads to the thinking that oxidatively stressed adipocytes release sEVs containing partially functional and oxidatively damaged mitochondria. The sEVs containing oxidized mitochondria are found to circulate to cardiac tissues, where they are taken up by cardiomyocytes, integrated into the cardiomyocyte mitochondrial network, and eventually degraded.

The release of selective mitochondrial proteins through adipocyte EVs suggests that this process may be facilitated by mitochondrial vesicles (MDVs). Cells have the ability to sort distinct mitochondrial proteins into either single- or double-membrane MDVs [55]. Single-membrane MDVs emerge from the mitochondrial outer membrane, carrying with them cargo proteins located on the outer mitochondrial membrane, such as translocase of the outer mitochondrial membrane 20 (TOMM20). On the other hand, double-membrane MDVs form through the simultaneous budding of both the mitochondrial inner and outer membranes, transporting inner mitochondrial membrane and matrix proteins, such as pyruvate dehydrogenase (PDH). Research has revealed that single-membrane MDVs form through membrane protrusion driven by the mitochondrial Rho 1/2 (MIRO1/2) complex, which is subsequently pinched off by dynamin-related protein 1 (DRP1) [56]. Two factors known to enhance mitochondrial ROS production, namely xanthine oxidase and the complex III inhibitor antimycin A, have been shown to increase MDV formation [3]. Indeed, in both cold-stressed BAT and adipocytes in FtMT-overexpressing mice, there is a simultaneous increase in mitochondrial ROS production and MDV formation [53, 54]. These findings support the notion that MDVs play a pivotal role in EV-mediated release of defective mitochondrial components by the adipose tissue.

#### Mito-release triggered by defects in intracellular mitochondrial quality control

Numerous intracellular pathways contribute to the maintenance of mitochondrial quality in mammalian cells. Two notable pathways involved in this process are mitophagy and the MDV pathway. These pathways play a crucial role in targeting defective mitochondrial components to the lysosomes for degradation, thereby ensuring the maintenance of mitochondrial activity.

Recent reports have indicated that when the lysosome-dependent elimination of mitochondria is hindered, cells tend to increase the production of EVs to remove mitochondria from the intracellular space. For instance, the formation of the protein-lipid conjugate Atg8-phosphatidylethanolamine (Atg8-PE) is essential for mitophagy. Therefore, in HeLa cells expressing Parkin but lacking Atg8-PE formation, inhibiting mitochondrial functions did not result in the clearance of damaged mitochondria through the mitophagy pathway. Instead, these cells eliminated damaged mitochondria by releasing them into the extracellular space [57]. Another example involves the expression of disease-promoting mutations in Optineurin (OPTN), a protein associated with mitophagy. Mutations in OPTN are known to cause impaired or aberrant mitophagy [58, 59]. In neurons expressing mutant OPTN, transmitophagy, a process involving the release of mitochondria, was activated [46]. Additionally, inhibiting lysosomal acidification in mouse embryonic fibroblasts resulted in an increased secretion of mitochondria within large extracellular vesicles [60]. Similarly, hearts from aged mice or individuals with Danon disease exhibited elevated levels of secreted EVs containing mitochondria [60]. Danon disease is caused by mutations in the lysosome-associated membrane protein 2 gene, which impairs autophagic degradation. Lastly, in neurons derived from Huntington's disease (HD) patients, damaged mitochondria were found to be eliminated through EV-mediated extracellular release [61]. Consistent with this finding, the levels of neuron-derived extracellular vesicles containing mitochondria were enhanced in biofluids of HD patients. This observation is linked to the compromised mitophagy pathway commonly observed in cellular models of HD [62].

Taken together, these findings suggest that mammalian cells activate the extracellular release of mitochondria as a compensatory mechanism when mitophagy is impaired. This highlights extracellular release as a mitochondrial quality control mechanism in mammalian cells.

## Conclusions

**Fig. 3:**
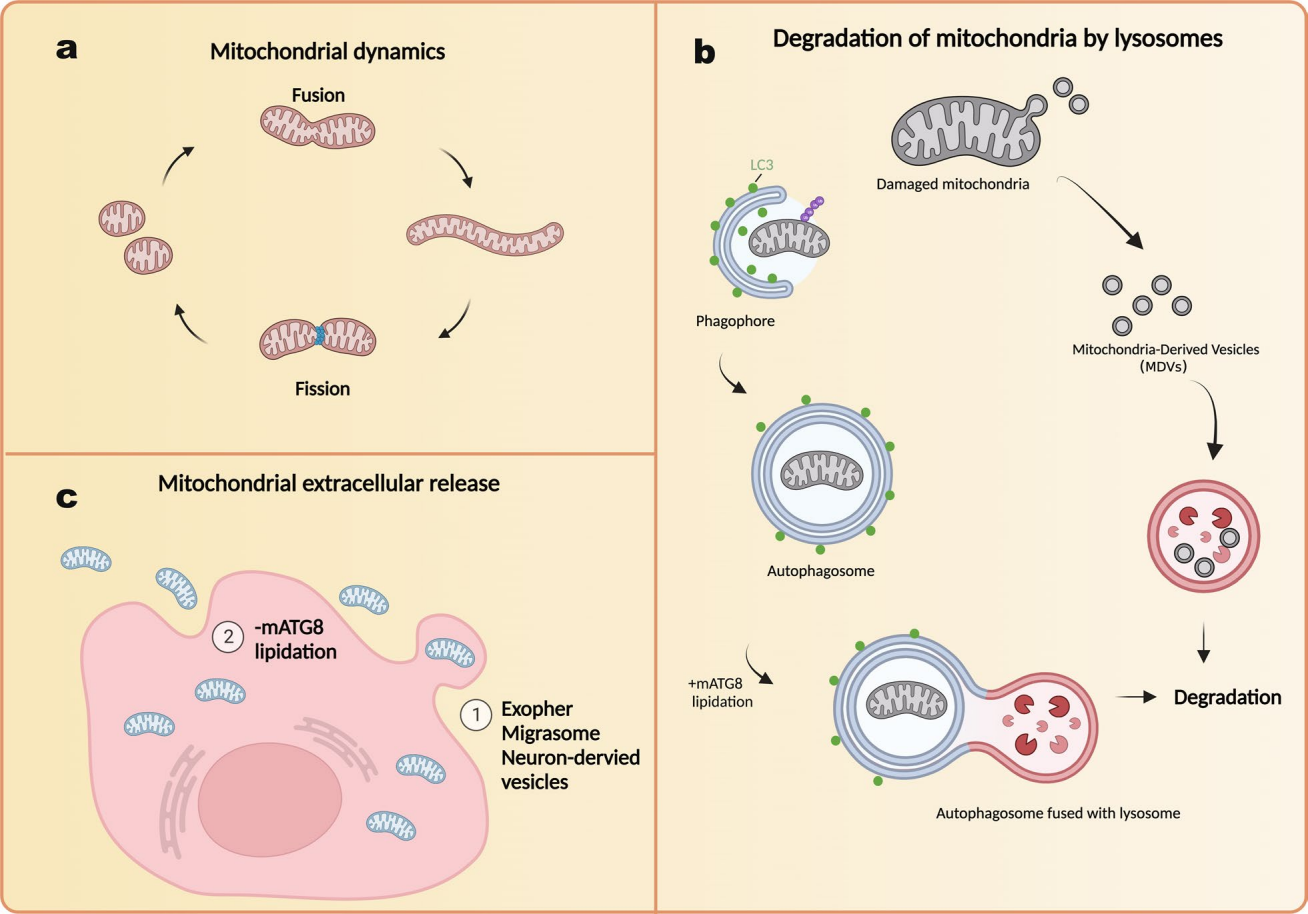
Mitochondrial Quality Control Pathways. Cells utilize a number of strategies to maintain mitochondria quality. **a**. Mitochondrial dynamics. A damaged segment of mitochondria splits off from healthy mitochondria. **b**. Lysosomal degradation of damaged mitochondria. Damaged mitochondrial fragments can be engulfed into phagophores, and delivered into lysosomes for degradation. This process is termed mitophagy. On the other hand, malfunctioning portion of mitochondria can be packed into small vesicles which bud off from mitochondria called mitochondria derived vesicles (MDVs). MDVs can be delivered to lysosomes and degraded. **c**. Mitochondrial extracellular release. (1) Damaged mitochondria can be removed through several extracellular release processes as shown in Fig. 2. (2) The lack of ATG8-PE on autophagosomes results in autophagosome-mediated mitochondrial release into the extracellular space. Figure created with BioRender.com

Mitochondrial quality control is essential for maintaining cellular energy production, regulating ROS production, preserving mitochondrial DNA integrity, and sustaining overall cellular homeostasis in mammalian cells. While intracellular pathways have been extensively studied and documented, recent findings highlighting the involvement of extracellular release in mitochondrial quality control demand further investigation to elucidate the underlying mechanisms. Additionally, it is important to determine how a cell's overall status influences its preference for using extracellular release as its quality control mechanism for mitochondria. Investigating whether cells that are more susceptible to mitochondrial stress or those with diminished autophagy capabilities tend to prioritize extracellular release as their primary mode of mitochondrial quality control is worth exploring. Examining the cellular consequences of defective extracellular release of dysfunctional mitochondria on cellular fitness will also be an area of substantial interest. Through these investigations, we will gain a better understanding of how mammalian cells prioritize between intracellular degradation and extracellular release for mitochondrial quality control (Fig. 3).

The discovery of extracellular release as a contributor to mitochondrial quality control poses new challenges for understanding these pathways. Cell imaging has been invaluable in elucidating intracellular mitochondrial quality control mechanisms like mitophagy and MDV-dependent pathways, where retained mitochondria can be easily tracked and quantified. However, monitoring extracellular release events presents difficulties, as released mitochondria can quickly move out of the focal plane. Recent studies have combined total internal reflection fluorescence microscopy and pH-sensitive fluorescent proteins to visualize the dynamics of EV release at the culture dish-cell interface [63]. The development of novel imaging platforms will be crucial for advancing our understanding of the extracellular release of mitochondria.

One intriguing pathway that requires further exploration for its involvement in mediating mitochondrial quality control is exophagy. Exophagy is a cellular process through which materials are expelled from the cytoplasm via amphisomes. Amphisomes are formed through the fusion of autophagosomes and multivesicular bodies, representing a convergence of the autophagic and endocytic pathways [64]. They carry cargo from autophagosomes and have the ability to fuse with the plasma membrane, releasing their contents into the extracellular space. It is well known that mitochondria can be targeted into autophagosomes. It is therefore plausible that cells use amphisomes to direct mitochondria toward either intracellular degradation or extracellular release. Indeed, several studies have reported autophagy-dependent release of mitochondria. In one study, mitochondria were observed to be loaded from the cytoplasm into LC3-containing vesicles, which migrated towards the cell periphery and became incorporated into outward budding blebs on the plasma membrane [37]. However, the observed outward budding blebs appeared distinct from a secretory mechanism involving amphisomes. In another study, it was found that mitochondria can be engulfed into LC3-free, autophagosome-like structures for secretion. However, it was again thought that secretion occurred independent of exophagy [57]. Determining whether exophagy plays a role in mammalian mitochondrial quality control requires further investigation.

Another aspect to consider in mitochondrial quality control, in addition to intracellular degradation and extracellular release, is the intercellular transportation of mitochondria. This process involves the transfer of mitochondria between cells without their release into the extracellular space [65]. Cellular structures such as tunneling nanotubes (TNTs) and gap junctions have been implicated in facilitating intercellular transport of functional mitochondria. TNTs are transient filamentous tubular membrane networks that connect cells and enable the transfer of various intracellular contents, including ions, proteins, bacteria, viruses, and organelles. Gap junctions, on the other hand, are specialized intercellular channels formed by connexin proteins that allow for direct communication and the exchange of small molecules and ions between adjacent cells. Investigating whether these structures also serve as potential routes for the transfer of damaged mitochondria will provide a more comprehensive understanding of their role in mitochondrial quality control.

The enormous progress made on the understanding of mitophagy has driven efforts in probing how autophagy similarly participates in the quality control of various cellular organelles within mammalian systems [1, 2, 66–73]. As emerging evidence suggests the involvement of extracellular release in mitochondrial quality control, it raises the intriguing possibility of whether extracellular release plays a more general role in organelle quality control. Recent studies have indicated that cells can release organelles other than mitochondria through EVs. Therefore, it would be valuable to investigate whether EV-dependent pathways universally regulate organelle quality.

## Data Availability

Not applicable.

## Abbreviations

ROS: Reactive oxygen species
EV: Extracellular vesicle
MVB: Multivesicular body
ILV: Intraluminal vesicle
6-OHDA: 6-Hydroxydopamine
CCCP: Carbonyl cyanide 3-chlorophenylhydrazone
TNT: Tunneling nanotubes
sPLA2-IIA: Secreted phospholipase A2 group IIA
MDV: Mitochondrial-derived vesicle
SERP: Secretory reticulophagy
MFG: Milk fat globule
PM: Plasma membrane
IFN: Interferon
TNF: Tumor necrosis factor
TUNEL assay: Terminal deoxynucleotidyl transferase dUTP nick end labeling assay
LPS: Lipopolysaccharide
OPTN: Optineurin
HD: Huntingtin’s disease
Atg8-PE: Atg8-phosphatidylethanolamine
cMacs: Cardiac-resident macrophages
MSCs: Mesenchymal stem cells
UV: Ultraviolet light
BAT: Brown adipose tissue
FtMT: Mitochondrial ferritin
VDAC: Voltage-dependent anion channel
HSP60: Heat shock protein 60
COXIV: And cytochrome c oxidase subunit 4l1
TOMM20: Translocase of outer mitochondrial membrane 20
PDH: Pyruvate dehydrogenase
PDHβ: Pyruvate dehydrogenase E1 subunit beta
MIRO1/2: Mitochondrial Rho 1/2
DRP1: Dynamin-related protein
